# Effects of different cooking methods on volatile flavor compounds, nutritional constituents, and antioxidant activities of *Clitocybe squamulosa*

**DOI:** 10.3389/fnut.2022.1017014

**Published:** 2022-10-21

**Authors:** Hui Yuan, Lijing Xu, Mingchang Chang, Junlong Meng, Cuiping Feng, Xueran Geng, Yanfen Cheng, Zongqi Liu

**Affiliations:** ^1^College of Food Science and Engineering, Shanxi Agricultural University, Taigu, China; ^2^Shanxi Key Laboratory of Edible Fungi for Loess Plateau, Taigu, China; ^3^Shanxi Engineering Research Center of Edible Fungi, Taigu, China

**Keywords:** *Clitocybe squamulosa*, cooking methods, volatile compounds, quality, antioxidant

## Abstract

To explore a scientific and reasonable cooking method for *Clitocybe squamulosa*, four cooking methods (boiling, steaming, microwaving, and frying) were applied to *C. squamulosa*, and the effects of different cooking methods on volatile flavor compounds, nutritional constituents, and antioxidant activities in *C. squamulosa* were systematically investigated. The results showed that 54, 53, 61, 63, and 49 volatile flavor compounds were detected in raw, boiled, steamed, microwaved, and fried samples, respectively. Large differences in volatile flavor compounds between the four cooking and raw samples were determined by using relative odor activity values (ROAV) and principal component analysis (PCA). In addition, steaming and microwaving could protect the nutrients of *C. squamulosa*, reduce losses during the cooking process and improve the color of cooked products compared to boiling and frying cooking methods. Meanwhile, cooking treatment exerted different effects on the antioxidant activity of *C. squamulosa*, and the antioxidant activity of *C. squamulosa* was the highest after microwave cooking treatment. This research can provide a theoretical basis for the cooking, processing and utilization of *C. squamulosa* and other wild edible fungi.

## Introduction

*Clitocybe squamulosa*, as a kind of “Yinpan” in “Taimo,” belongs to the family Tricholomataceae and genus *Clitocyb*. It is widely eaten as a wild edible fungus with medicinal and edible homology. It is one of the famous specialties of Shanxi Province, China, mainly growing on the ground in the mix of pine, spruce, and birch in Wutai Mountain, Xinzhou (1). Its fruiting bodies are rich in protein, amino acids, carbohydrates, minerals, and a variety of bioactive compounds, including polysaccharides, polyphenols, and flavonoids (2). Bioactive compounds have antioxidant (3, 4), antitumor (5), anti-inflammatory (6), hypoglycemic and hypolipidemic (7, 8) functions have been extensively reported. In addition, the sensory characteristics of edible fungi have also attracted attention. *C. squamulosa* has a unique flavor and is deeply loved by local residents. In recent years, with the promotion of the concept of “one meat, one vegetable, and one mushroom,” the market demand for edible fungi has increased rapidly (9). *C. squamulosa*, as a delicacy, has important cultural and commercial values. It is noteworthy that eating wild edible fungi without heating may result in serious safety hazards to human beings, and edible fungi must undergo different cooking treatments before people can eat them safely (10, 11), making it important to study the effect of cooking treatment on the quality of *C. squamulosa*.

China has a long cooking history and culture, including various cooking methods such as boiling, steaming, frying, grilling, smoking, etc. In recent years, with the fusion of Chinese and Western food cultures, new cooking methods have gradually emerged in the domestic market, such as microwave, vacuum low-temperature, and high-pressure cooking (12). Cooking plays an essential role in dietary quality attributes by not only disinfecting and sterilizing and improving the nutritional value but also improving the flavor, color and texture of food (13). However, the heat-transfer medium, cooking time, and cooking temperature of the cooking method in the ripening process are different, which will lead to different degrees of changes in the color, texture, flavor, and nutrients of food materials (14). These are closely related to the edible value of edible fungi. For instance, Liu et al. (15) evaluated the effects of four domestic cooking methods on the bioavailability of nutrients and antioxidant activity of *Oudemansiella radicata*. The results showed that when *O. radicata* was used as food material, steaming and microwaving which can improve the edible value of *O. radicata* to a greater extent were recommended. Mena García et al. (16) discovered that the phenolic compound concentrations of wild *Boletus edulis* mushroom were significantly decreased in all cooking treatments (confit, grill, roast, and marinade), and the antioxidant activities of DPPH and FRAP were reduced. In addition, the flavor, nutritional characteristics, and antioxidant activity of mushrooms may vary with the varieties and thermostability. For example, Selli et al. (17) found that the volatiles and key odorants of two edible fungi (*Agaricus bisporus* and *Pleurotus ostreatus*) differed significantly depending upon cooking method (boiling and oven cooking). Ng and Tan (18) studied the changes of *in vitro* antioxidant activity of five edible fungi (*A. bisporus*, *Flammulina velutipes*, *Lentinula edodes*, *P. ostreatus*, and *Pleurotus eryngii*) after being treated with different cooking methods (boiling, microwave, steam, and pressure). Among them, the antioxidant activity of pressure-cooked *P. eryngii* was the highest. In the meanwhile, the researchers further explored whether the antioxidant activity was significantly different due to the different types of mushrooms and cooking methods. The results showed that microwaving *A. bisporus* and *F. velutipes*, boiling *Auricularia polytricha*, and pressure-cooking *L. edodes* and *Pleurotus sajor-caju* yielded the best antioxidant activities (19). However, there remains a lack of systematic research into the effects of different cooking methods on the volatile flavor, nutritional content, and antioxidant activity of *C. squamulosa*.

Therefore, to find the scientific and reasonable processing and cooking methods of mushrooms, by taking *C. squamulosa* as the research object, the effects of boiling, steaming, microwaving, and frying on the flavor, color, nutrients, and antioxidant activity of *C. squamulosa* were compared. The purpose of this study was to select the most suitable cooking method for *C. squamulosa* and to provide a theoretical basis for the cooking, processing, and utilization of wild edible fungi.

## Materials and methods

### Materials

*Clitocybe squamulosa* was selected from Wutai Mountain in Shanxi Province, China, and provided by the Shanxi Edible Fungi Engineering Technology Research Center. *C. squamulosa* was identified by Hu and Wang (1, 2, 20, 21), and the complete fruiting body was selected as the experimental sample. *C. squamulosa* was washed with paper towels and freeze-dried for further processing in this study, before being randomly divided into five groups: raw and four cooking samples.

### Cooking methods

Four different cooking methods were tested: boiling, steaming, microwaving, and frying (15).

Boiling: 50 g *C. squamulosa* could be boiled in 500 mL of boiling water on a hot plate at 100°C for 6 min.

Steaming: *C. squamulosa* (50 g) was placed into a steamer and steamed for 6 min at 100°C.

Microwaving: 50 g *C. squamulosa* was placed into a porcelain bowl filled with 100 mL of water and microwaved at 800 W for 2 min.

Frying: first, *C. squamulosa* could be placed in water at 37°C for 1 min, removed, and filter paper was used to absorb the excess water. Then, 50 mL of soybean oil was heated to 180°C in a temperature-controlled frying pan. Finally, 50 g *C. squamulosa* was added and fried for 2 min.

All cooking experiments were performed in triplicate. All samples were drained of water or oil on the surface with filter paper. At the same time, vacuum freeze drying was conducted before the determination of nutrient components and activity to prevent water interference in the determination.

### Volatile flavor compound analysis

#### Headspace solid-phase microextraction-gas chromatography-mass spectrometry analysis

The volatile flavor compounds of the raw and cooked samples of *C. squamulosa* were determined by headspace solid-phase microextraction (HS-SPME) (Stable Flex, Supelco, Bellefonte, PA, USA) combined with gas chromatography-mass spectrometry (GC-MS) (Trace ISQ, Thermo Fisher Scientific, Waltham, MA, USA) (22). The specific steps in these operations are described as follows:

Method: all samples were weighed, placed into 10 mL headspace bottles and heated at 80°C for 30 min. Then, the extraction head was pulled back and quickly inserted into the GC-MS injection port. The volatiles in *C. squamulosa* were desorbed at 250°C for 5 min and GC-MS determination and analysis.

Gas chromatography-mass spectrometry analysis: the column used was a TG-5MS capillary column (30 m × 0.25 mm × 0.25 μm), with high purity helium (99.999%) as the carrier. The flow rate was 1.0 mL/min and the sample was injected without partial flow. The initial column temperature was maintained at 35°C for 3 min and then increased to 180°C at 5°C/min with a 3 min hold time. Finally, it was raised to 250°C at 10°C/min with a 5 min hold time. The MS electronic energy was 70 eV, the transmission line temperature was 250°C, the quadrupole temperature was 150°C, and the mass scanning range was 35 ≤ *m*/*z* ≤ 500.

The chemical components were determined by searching the NIST W8N08L standard spectral library, the retention index (RI) was determined using the C_5_-C_30_ n-alkanes (Sigma-Aldrich, Louis, MO, USA) as the standard, and the relative content of each aroma component was obtained according to the peak area normalization method.

#### Determination of relative odor activity values

The relative odor activity value (ROAV) method determines the key volatile flavor compounds in *C. squamulosa* (23). The ROAV_*max*_ of the compound that contributes the most to the overall flavor of the sample is defined as 100, and the ROAV of other compounds are calculated according to the following formula:


$$R\,O\,A\,V_{i}\approx \frac{C_{i}}{C_{m\,a\,x}}\times \frac{T_{m\,a\,x}}{T_{i}}\times 100$$

where *C*_*i*_ and *T*_*i*_ are the relative percentage and odor threshold of each flavor compound, respectively; *C*_*max*_ and *T*_*max*_ denote the relative percentage and odor threshold of the flavor compound that contributes the most to the overall flavor, respectively. All components satisfy 0 < ROAV ≤ 100, and the larger the ROAV, the greater the component contribution to the overall flavor of the *C. squamulosa* samples. In the case of the ROAV ≥ 1, the compounds significantly affect the overall flavor of *C. squamulosa* and are considered as key volatile flavor compounds; when 0.1 ≤ ROAV < 1, the compounds exert a modifying effect on the overall flavor of *C. squamulosa*.

### Color measurement

The changes in color between raw and cooked samples were evaluated by an automatic colorimeter (ColorQuest XE, HunterLab, Shanghai, China) (24). Standard whiteboards and blackboards were used for calibration, and nine sites on the pileus surface were randomly selected to measure and record the values of L*, a*, and b*, representing lightness, greenness/redness, and blueness/yellowness, respectively. Then the hue angle (*H*), a/b values (*h*), chroma (C*), and total color difference (Δ*E*) were calculated according to the following formula:


Δ⁢E=(Δ⁢L*+Δ⁢a*+Δ⁢b*)12⁢C*=(a2*+b2*)12

H=a⁢r⁢c⁢t⁢a⁢n⁢b*a* h=ab

### Conventional nutrient analysis

The composition of ash, water, protein, and fat was analyzed according to standard official analysis methods (AOAC) (25). Then, the total carbohydrates and total energy were calculated based on the following formula (15):


Total⁢carbohydrates⁢(g)=⁢100-(g⁢ash+g⁢water+g⁢crude⁢protein+g⁢crude⁢fat)

Total⁢energy⁢(kJ)=⁢17×(g⁢crude⁢protein+g⁢total⁢carbohydrates)+ 37×(g⁢crude⁢fat)

### Amino acid analysis

Amino acids (AAS) in all samples were extracted following the method described in the Association of Official Analytical Chemists procedures (AOAC). The hydrolyzed amino acid (HAAS) contents of raw and cooked samples were determined using an amino acid auto-analyzer (Biochrom, Cambridge, UK) (26).

### Bioactive compounds analysis

#### Polysaccharide content determination

The polysaccharide was extracted according to the method described by Guo et al. (2). The polysaccharide content was determined by the phenol-sulfuric acid method and using glucose (0–0.15 mg/mL) as a standard. The polysaccharide content was expressed as milligrams of glucose equivalent per gram mass of dry sample.

#### Total phenolic content determination

The total phenolic was extracted according to the method described by Deng et al. (27). The total phenolic content was determined by the Folin–Ciocalteu method and using gallic acid (0–600 μg/mL) as a standard. The total phenolic content was expressed as milligrams of gallic acid equivalent per gram mass of dry samples.

#### Flavonoid content determination

The flavonoid was extracted according to the method described by Liu et al. (28). The flavonoid content was determined by the sodium nitrite-aluminum nitrate colorimetric method and using rutin (0–2.5 μg/mL) as a standard (29). The flavonoid content was expressed as milligrams of rutin equivalent per gram mass of dry sample.

### Antioxidant activities

#### Preparation of aqueous extract

The aqueous extract was prepared following the procedure described by Guo et al. (21) and Sharpe et al. (30) with some modifications. Briefly, all samples were pulverized and mixed with ultrapure water at a ratio of 1:30 (*w/v*). The mixture was extracted after 3.6 h in a water bath at 80°C and centrifuged at 5,000 rpm for 10 min, and the supernatant was collected. The obtained filtrate was concentrated on a rotary evaporator (IKA, China). Finally, vacuum freeze drying was performed. The dry samples were stored at 4°C for further analysis.

#### Determination of antioxidant activities

The DPPH, ABTS, and OH radical scavenging abilities of all samples were determined according to the methods of Pan et al. (31). The extracts were prepared into different mass concentration gradients (0.25, 0.5, 1, 2, and 4 mg/mL). The DPPH radical scavenging abilities of raw and cooked samples were determined according to the method of the DPPH Kit (Solarbio Science, Beijing, China). In short, the sample solution (0.25, 0.5, 1, 2, and 4 mg/ml) and reaction reagent were added to the 96-well plate in sequence, vortexed, and incubated at room temperature in the dark for 30 min, and the absorbance was measured at 515 nm. The ABTS radical scavenging abilities of raw and cooked samples were determined according to the method of the ABTS kit (Solarbio Science, Beijing, China). Briefly, 50 μL of sample solutions (0.25, 0.5, 1, 2, and 4 mg/mL) were added and then reaction reagent was added to the 96-well plate. After full mixing, the mixture was incubated at room temperature in the dark for 6 min, and the absorbance was measured at 405 nm. The OH radical scavenging abilities of raw and cooked samples were determined according to the method of the OH Kit (Solarbio Science, Beijing, China). Briefly, the reaction reagents were added to the 96-well plate in turn, vortexed, and placed in a constant temperature incubator at 37°C for 60 min, centrifuged at 10,000 rpm for 10 min, and the absorbance was measured at 536 nm. Notably, ascorbic acid (Vc) was used as a positive control for the DPPH, ABTS, and OH radical scavenging methods and the half inhibitory concentration (IC_50_) values for scavenging DPPH, ABTS, and OH radicals were calculated by linear regression analysis. A higher IC_50_ value corresponds to a lower antioxidant activity.

### Statistical analysis

Statistical analysis was conducted using the SPSS 20.0 software (IBM Inc., Chicago, IL, USA). All data are expressed as the mean ± standard deviation (SD) and were analyzed by one-way analysis of variance (ANOVA). Differences were considered significant at *p* < 0.05. Principal component analysis (PCA) was performed using JMP Pro 16.

## Results and discussion

### Effects of cooking methods on volatile flavor compounds of *Clitocybe squamulosa*

#### Headspace solid-phase microextraction-gas chromatography-mass spectrometry analysis of *Clitocybe squamulosa* with different cooking methods

Table 1 lists the volatile flavor compounds identified using HS-SPME-GC-MS to study the effects of different cooking methods on volatile components of *C. squamulosa*. A total of 128 volatile components were identified in all samples, including alcohols, aldehydes, ketones, esters, acids, alkanes, heterocyclic, aromatic hydrocarbons, ethers, and others. Moreover, a total of 54, 53, 61, 63, and 49 volatile components were detected in raw, boiled, steamed, microwaved, and fried cooking samples, respectively, of which alcohols, aldehydes, esters, and ketones were the major chemical classes.

**TABLE 1:** Relative contents of volatile compounds in *C. squamulosa* with different cooking methods.

	English names	RI	Molecular formula	Relative percentage (%)				
				
				CS	BCS	SCS	MCS	FCS
Alcohol	Ethanol	–	C_2_H_6_O	–	–	–	–	23.9
	2,3-Butanediol	–	C_4_H_10_O_2_	–	–	–	–	0.2
	3-Methyl-1-butanol	708	C_5_H_12_O	0.36	–	–	–	–
	1-Pentanol	750	C_5_H_12_O	–	–	0.2	0.15	–
	Hexyl alcohol	799	C_6_H_14_O	0.21	–	–	–	–
	1,2,6-hexanetriol	834	C_6_H_14_O_3_	–	–	0.14	–	0.6
	2-Methylcyclohexanol	902	C_7_H_14_O	–	–	–	0.2	–
	1-Octen-3-ol	982	C_8_H_16_O	1.15	25.35	2.15	2.54	32.99
	Phenethyl alcohol	989	C_8_H_10_O	4.68	0.53	0.65	0.67	0.26
	3-Heptanol, 6-methyl-	997	C_8_H_18_O	–	–	–	–	0.16
	2-Heptanol, 6-methyl-	1024	C_8_H_18_O	–	–	1.6	–	–
	2-Octyn-1-ol	1032	C_8_H_14_O	–	0.69	0.51	0.38	–
	(Z)-5-Octen-1-ol	1036	C_8_H_16_O	–	–	0.2	–	–
	5-Octyn-4-ol, 2-methyl-	1097	C_9_H_16_O	–	–	–	0.51	–
	*Trans*-2-Octen-1-ol	1100	C_8_H_16_O	–	2.6	–	–	3.9
	Alpha-Terpineol	1187	C_10_H_18_O	–	–	0.68	–	–
	Rac-Isopinocampheol	1196	C_10_H_18_O	–	–	0.81	–	–
	2-propyl-1-Heptanol	1197	C_10_H_22_O	–	–	–	0.2	–
	2,4-Decadien-1-ol	1230	C_10_H_18_O	–	–	–	–	0.38
	2-Undecen-1-ol, (2E)-	1298	C_11_H_22_O	–	–	–	–	0.7
	1-Octanol, 2-butyl-	1460	C_12_H_26_O	0.8	0.11	0.27	0.13	–
	1-Tridecanol	1523	C_13_H_28_O	–	–	0.15	–	0.6
	Nonadecanol	1698	C_19_H_40_O	–	0.11	0.17	–	–
	Isophytol	1826	C_20_H_40_O	–	–	0.3	0.19	–
	11-Hexadecyn-1-ol	1853	C_16_H_30_O	–	–	0.22	–	–
	Hexadecanol	1860	C_16_H_34_O_4_	–	0.29	–	–	–
Aldehyde	Butanedial	–	C_4_H_6_O_2_	–	–	–	–	0.5
	Isovaleraldehyde	735	C_5_H_10_O	–	0.15	–	–	0.21
	Valeraldehyde	802	C_5_H_10_O	0.15	0.28	0.42	0.19	0.2
	Hexanal	867	C_6_H_12_O	0.81	2.76	3.31	1.92	1.57
	Hexanal, 2-ethyl-	898	C_8_H_16_O	–	–	–	0.15	–
	Methyl valeraldehyde	902	C_6_H_12_O	–	0.12	–	0.13	–
	Decanal	902	C_10_H_20_O	–	0.97	0.72	0.46	0.14
	Heptaldehyde	953	C_7_H_14_O	–	0.47	0.29	0.21	0.9
	(E)-2-Octenal	979	C_8_H_14_O	0.24	0.39	0.27	0.23	0.22
	octanal	995	C_8_H_16_O	–	0.93	–	0.43	0.55
	Benzaldehyde	1004	C_7_H_6_O	–	0.44	–	0.37	0.39
	(E)-2-Nonen-1-al	1068	C_9_H_16_O	–	0.17	0.15	–	2.5
	1-Nonanal	1112	C_9_H_18_O	–	2.86	1.53	1.44	–
	Undecan-4-olide	1306	C_11_H_20_O_2_	0.2	–	–	–	–
Ketones	2-Pyrrolidinone	–	C_4_H_7_NO	1.15	–	1.84	1.13	–
	2H-Pyran-2-one, 5,6-dihydro-	734	C_5_H_6_O_2_	1.66	2.48	2.4	1.66	0.82
	2-Heptanone	948	C_7_H_14_O	0.61	2.25	4.3	3.25	–
	3-Octanone	981	C_8_H_16_O	6.91	0.87	1.41	1.82	0.44
	3-Octen-2-one	991	C_8_H_14_O	0.46	0.68	1.9	0.69	–
	1-Octen-3-one	998	C_8_H_14_O	2.25	2.47	4.74	3.8	0.9
	6-Undecanone	1321	C_11_H_22_O	0.21	–	–	–	–
	2-Undecanone	1328	C_11_H_22_O	2.86	1.26	1.64	–	–
	2-Dodecanone	1432	C_12_H_24_O	0.32	0.17	0.24	0.2	–
	Methanone, dicyclohexyl-	1513	C_13_H_22_O	–	–	0.75	0.26	0.5
	Geranylacetone	1524	C_13_H_22_O	–	0.39	0.31	1.42	0.12
Ester	Butanoic acid, 4-hydroxy-	–	C_4_H_8_O_3_	–	0.17	0.35	–	0.54
	γ-Butyrolactone	–	C_4_H_6_O_2_	–	–	–	0.2	–
	δ-Valerolactone	689	C_5_H_8_O_2_	0.83	–	–	0.11	–
	γ-Valerolactone	694	C_5_H_8_O_2_	0.16	–	–	–	0.8
	Isoamyl nitrite	715	C_5_H_11_NO_2_		–	–	0.15	0.17
	γ-Valerolactone	752	C_5_H_8_O_2_	0.86	–	–	–	–
	4-Hexanolide	792	C_6_H_10_O_2_	1.23	–	–	–	–
	DL-sec-Butyl acetate	814	C_6_H_12_O_2_	0.17	–	–	–	–
	Dihydro-3-methyl-2(3H)-Furanone	840	C_5_H_8_O_2_	0.19	–	–	–	–
	Isobutyl acetate	845	C_6_H_12_O_2_	0.31	–	–	–	–
	Pentanoic acid, 2- methyl-, methyl ester	933	C_7_H_14_O_2_	0.35	–	0.15	–	0.5
	Banana oil	941	C_7_H_14_O_2_	1.59	–	–	0.2	–
	Isoamyl acetate	963	C_7_H_14_O_2_	2.31	–	–	0.39	–
	Hexyl acetate	1018	C_8_H_16_O_2_	2.46	–	–	0.18	–
	γ-Nonanolactone	1087	C_9_H_16_O_2_	3.53	1.5	1.43	1.25	–
	Phenethyl acetate	1164	C_10_H_12_O_2_	2.13	–	–	–	–
	Ethyl caprylate	1179	C_10_H_20_O_2_	0.64	0.33	–	–	0.1
	3-octyl acetate	1182	C_10_H_20_O_2_	1.3	–	–	–	–
	Isobutyl hexanoate	1186	C_10_H_20_O_2_	0.2	–	–	0.14	–
	Hexanoic acid, pentyl ester	1276	C_11_H_22_O_2_	–	–	–	0.13	–
	Isopentyl hexanoate	1286	C_11_H_22_O_2_	0.66	0.24	0.46	0.56	–
	Hexanoic acid, 2-methylbutyl ester	1329	C_11_H_22_O_2_	0.22	–	–	0.19	–
	Isovaleric acid, phenethyl ester	1498	C_13_H_18_O_2_	–	0.21	–	–	–
	Butanoic acid, 2-ethylhexyl ester	1560	C_12_H_24_O_2_	–	–	–	–	0.8
	Geranyl 3-methylbutanoate	1725	C_15_H_26_O_2_	0.16	–	–	–	–
	2,2,4-trimethyl-1,3-pentanediol diisobutyrate	1867	C_16_H_30_O_4_	–	0.27	0.8	0.14	–
Acids	Glycine, L-alanyl-	–	C_2_H_7_NO_2_	–	0.25	0.4	0.27	0.34
	DL-Homocysteine	–	C_4_H_9_NO_2_S	–	–	3.7	–	–
	Isobutyric acid	–	C_4_H_8_O_2_	–	–	–	–	0.1
	Isovaleric acid	734	C_5_H_10_O_2_	0.46	0.31	0.46	0.23	0.21
	DL-2-Methylbutyric acid	736	C_5_H_10_O_2_	–	–	0.17	–	–
	Acetic acid glacia	835	C_2_H_4_O_2_	3.32	–	4.57	2.38	4.71
	2-Methylhexanoic acid	964	C_7_H_14_O_2_	–	–	–	0.14	–
	4-Methyloctanoic acid	1126	C_9_H_18_O_2_	–	–	0.19	–	–
Alkanes	Hexane	600	C_6_H_14_	–	–	3.2	1.46	–
	1,3- dimethyl-, *cis*-Cyclopentane	700	C_7_H_14_	1.13	–	–	–	–
	1-Chlorohexane	845	C_6_H_13_Cl	–	–	–	0.24	–
	Octane, 3,5-dimethyl-	1000	C_10_H_22_	0.13	–	–	0.45	–
	Undecane	1100	C_11_H_24_	0.65	0.29	0.42	1.41	0.48
	Dodecane	1200	C_12_H_26_	2.39	1.32	1.23	1.6	0.97
	Decane, 2-methyl-	1200	C_12_H_26_	–	–	0.15	0.21	–
	Tetradecane	1400	C_14_H_30_	4.3	1.67	3.8	1.29	0.65
	Tridecane, 3-methylene-	1400	C_14_H_28_	–	0.21	–	–	–
	Undecane, 3-methyl-	1458	C_12_H_24_O	–	0.35	0.28	–	0.25
	Pentadecane	1500	C_15_H_32_	1.29	–	–	–	–
	Hexadecane	1600	C_16_H_34_	–	1.13	0.93	0.99	0.3
	Heptadecane	1700	C_17_H_36_	–	0.43	–	–	–
	Oxirane, 2-tetradecyl-	1861	C_16_H_32_O	–	0.27	–	–	0.6
	Heneicosane	2100	C_21_H_44_	0.4	–	–	–	–
	Heptacosane	2700	C_27_H_56_	–	–	0.28	–	–
Pyrazines	2,6-Dimethylpyrazine	843	C_6_H_8_N_2_	4.16	–	0.33	0.38	–
	2,5-Dimethyl pyrazine	843	C_6_H_8_N_2_	–	0.25	–	–	–
	2-Ethyl-5-methylpyrazine	954	C_7_H_10_N_2_	0.68	–	–	–	–
	2,3,5-Trimethylpyrazine	954	C_7_H_10_N_2_	–	–	–	0.76	–
	Pyrazine,2-ethyl-3,5-dimethyl-	1031	C_8_H_12_N_2_	0.49	–	–	–	–
	Pyrazine,2-butyl-3-methyl-	1098	C_9_H_14_N_2_	0.44	–	–	0.11	–
Aromatic	Ethylbenzene	890	C_8_H_10_	–	0.22	0.25	–	–
	1,4-Xylene	921	C_8_H_10_	–	0.22	–	0.15	0.44
	1,3-Xylene	925	C_8_H_10_	–	–	0.49	–	–
	Ethylbenzene	926	C_8_H_10_	–	–	–	0.13	0.34
	1,3,5,7-cyclooctatetraene	964	C_8_H_8_	–	–	–	–	1.15
	Naphthalene	1196	C_10_H_8_	0.16	0.33	0.42	0.32	0.11
	1-isopropyl-2-methylbenzene	1209	C_10_H_14_	–	–	0.17	–	0.7
	7H-Benzocycloheptene	1280	C_11_H_10_	–	–	0.17	0.11	–
	7-Tetradecene	1598	C_14_H_28_	–	–	0.23	–	–
Ethers	Ethene, (2-methoxyethoxy)-	636	C_5_H_10_O_2_	–	4.49	–	–	–
	N-Hexyl n-octyl ether	1631	C_14_H_30_O	–	–	–	–	0.6
	1,1′-oxybis-Decane	1793	C_20_H_42_O	0.87	–	–	–	–
	Octane, 1,1′-oxybis-	1877	C_16_H_34_O	–	–	0.15	–	–
Other	Lenthionine	–	C_2_H_4_S_5_	–	0.27	–	–	0.15
	Trimethylamine	–	C_3_H_9_N	–	–	2.54	2.4	–
	Ammonium acetate	–	C_2_H_7_NO_2_	–	3.33	–	–	–
	Hexanenitrile	831	C_6_H_11_N	–	0.11	0.27	–	—
	1H-Pyrrole,1,2,5-trimethyl-	934	C_7_H_11_N	0.5	–	–	0.12	–
	2-Pentylfuran	1102	C_9_H_14_O	3.6	5.2	9.37	3.93	1.26
	2-Dodecanone	1496	C_12_H_24_O	–	0.17	–	0.2	–
	2a,3a-Epoxy-5a-androstan-17b-ol	1706	C_19_H_30_O_2_	–	0.12	–	–	0.7

CS, *C. squamulosa*; BCS, boiling-cooked *C. squamulosa*; SCS, steaming-cooked *C. squamulosa*; MCS, microwaving-cooked *C. squamulosa*; FCS, frying-cooked *C. squamulosa*.

Cooking methods altered the volatile flavor profiles of *C. squamulosa*. Briefly, esters were abundant in the saw sample, but large losses occurred during the cooking process. This may be attributed to the conversion of ester compounds into other substances as the result of thermal decomposition or the action of enzymes in the cooking process, such as degrading esters and producing alcohols and acids compounds (32). In addition, different cooking methods had different effects on alcohols, aldehydes and ketones in *C. squamulosa*. During high-temperature ripening, these compounds will be lost, but many new compounds will also be produced. This series of changes can be caused by the Maillard reaction, the interaction of amino acids or proteins with oxidized lipids, and the degradation of long-chain compounds during heating (22). Therefore, the flavors of *C. squamulosa* processed by different cooking methods are different, due to the comprehensive effect of a variety of volatile flavor compounds with different characteristics.

Alcohols, with a mild characteristic odor, are important volatile compounds in mushrooms (22). Among alcohols, 1-octene-3-ol is the key volatile component of edible fungi, also known as “mushroom alcohol,” which is mainly derived from the reaction of lipoxygenases in polyunsaturated fatty acids, and its stability is poor (33). In contrast, boiling and frying cooking have the greatest effect on alcohols, and the relative contents of 1-octene-3-ol in boiled and fried samples were 22.02 times and 28.69 times that of the raw sample, respectively. In addition, a large amount of ethanol is produced in the fried cooking sample, which has a mellow aroma and flavor.

Aldehydes and ketones are mainly derived from polyunsaturated fatty acid oxidation, amino acid degradation, and the Maillard reaction (2, 34). Due to the low odor threshold, they are more critical volatile compounds than alcohols, which will strongly affect the aroma of mushroom products. It is worth noting that all cooked samples show an increase in the content of aldehydes. The contents of hexanal, 1-nonanal and decanal are high. Among them, hexanal has the flavor of grass, 1-nonanal shows the flavor of rose and citrus and decanal has the aroma of fat (16), which increases the complexity of mushroom aroma. The relative contents of 1-nonanal and decanal in boiling samples are higher, and the content of hexanal in cooked samples is greater. The threshold of ketone is higher than that of aldehyde. Among the four cooking methods, microwaved samples retain ketones better and are more conducive to the formation of ketones. The contents of 1-octene-3-one, 2-heptanone, 2h-pyran-2-one, 5,6-dihydrogen, 3-octene-2-one, and 2-pyrrolidone are higher.

#### Key aroma component analysis of *Clitocybe squamulosa* after different cooking methods

The contribution of volatile flavor compounds to the overall aroma of the sample depends on the concentration and threshold (23). Through searching, a total of 33 volatile flavor components of aroma thresholds were found. ROAV values were calculated through the concentrations and thresholds of volatile flavor compounds (Table 2). In the raw samples, 10 compounds were screened out as the key volatile flavor components of *C. squamulosa*. After cooking, the boiled, steamed, microwaved, and fried samples contained 8, 17, 14, and 3 key flavor compounds, respectively. It is the differences between these flavor compounds that form their own unique volatile flavors under different cooking methods. Notably, in raw samples, 2,6-dimethylpyrazine was defined as the component that contributed the most to the overall flavor. However, among all the cooked samples, 1-octen-3-ol was defined as the component that contributed the most to the overall flavor. 1-octen-3-ol mainly has a mushroom flavor, so the mushroom flavor of *C. squamulosa* is better released after cooking. In addition, key flavor compounds were significantly increased after steaming and microwave cooking, especially aldehydes, including valeraldehyde, hexanal, (E)-2-octenal, benzaldehyde, octanal, 1-nonanal, (E)-2-nonen-1-al, decanal, and heptaldehyde, which mainly have fruit, almond, fat, and grass flavors (16).

**TABLE 2:** Key volatile compounds in *C. squamulosa* with different cooking methods.

English names	Odor threshold (μ g/kg)	ROAV				
		
		CS	BCS	SCS	MCS	FCS
1-Octen-3-ol	1.00	47.55	100.00	100.00	100.00	100.00
Hexyl alcohol	5.60	1.55	–	–	—	–
Phenethyl alcohol	1000.00	0.19	0.00	0.03	0.03	0.00
1-Pentanol	5.00	–	–	1.86	1.18	–
Ethanol	100,000.00	–	–	–	–	0.00
Valeraldehyde	12	0.52	0.09	1.63	0.62	0.05
Hexanal	4.50	7.44	2.42	34.21	16.80	1.06
(E)-2-Octenal	3.00	3.31	0.51	4.19	3.02	0.22
Isovaleraldehyde	9	–	0.07	–	–	0.07
Benzaldehyde	3.00	–	0.58	–	4.86	0.39
octanal	3.40	–	1.08	–	4.98	0.49
1-Nonanal	1.10	–	10.26	64.69	51.54	–
(E)-2-Nonen-1-al	0.19	–	3.53	36.72	–	39.88
Decanal	3	–	1.28	11.16	6.04	0.14
Heptaldehyde	3.00	–	–	4.50	–	0.52
Butanedial	7.00	–	–	–	–	0.22
2-Heptanone	140	0.18	0.06	1.43	0.91	–
1-Octen-3-one	5.00	18.61	1.95	44.09	29.92	0.55
3-Octanone	28.00	10.20	0.12	2.34	2.56	0.05
2-Undecanone	7.00	16.89	0.71	10.90	–	–
Geranylacetone	10	–	0.15	1.44	5.59	0.04
Isobutyl acetate	66.00	0.19	–	–	–	–
Acetic acid glacia	22000.00	0.01	–	0.01	0.00	0.00
Isovaleric acid	400.00	0.05	0.00	0.05	0.02	0.00
Isobutyric acid	4100.00	–	–	–	–	0.00
Hexane	580.00	–	–	0.26	0.10	–
2-Methylpyrazine	60.00	0.21	–	–	–	–
2,6-Dimethylpyrazine	1.72	100.00	–	8.92	8.70	–
2-Ethyl-5-methylpyrazine	16.00	1.76	–	–	–	–
2,5-Dimethyl pyrazine	1.82	–	0.54	–	–	–
2,3,5-Trimethylpyrazine	400	–	–	–	0.07	–
2-Pentylfuran	6.00	24.81	3.42	72.64	25.79	0.64
Trimethylamine	2.40	–	–	49.22	39.37	–

CS, *C. squamulosa*; BCS, boiling-cooked *C. squamulosa*; SCS, steaming-cooked *C. squamulosa*; MCS, microwaving-cooked *C. squamulosa*; FCS, frying-cooked *C. squamulosa*. The odor thresholds were referenced from a book named odor threshold compilations of odor threshold values in air, water and other media (second enlarged and revised edition) or taken from references (23, 50).

#### Principal component analysis of volatile components

**FIGURE 1:**
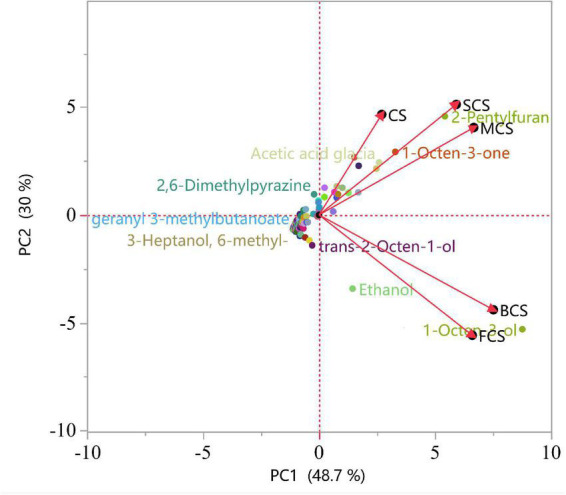
Principal component analysis (PCA) scores and load plots of volatile flavor compounds under different cooking methods. CS, *C. squamulosa*; BCS, boiling-cooked *C. squamulosa*; SCS, steaming-cooked *C. squamulosa*; MCS, microwaving-cooked *C. squamulosa*; FCS, frying-cooked *C. squamulosa*.

Principal component analysis was performed to visualize the underlying relationships between raw and four cooked samples. As shown in Figure 1, PC1 and PC2 accounted for 78.7% of the total variance. The score plot clearly compares the differences between raw and cooked samples. In addition, load plots were also performed to estimate the differences between samples. Samples could be divided into two groups: one represents the raw samples, steamed samples, and microwaved samples, and the other represents the boiled samples and fried samples. These results showed that the volatile components of *C. squamulosa* changed after cooking, and the choice of cooking technique exerted varying degrees of influence on thereon. Compared with the other two cooking methods, steaming and microwaving cooked can retain the original flavor and give unique flavor.

### Effects of cooking methods on the color of *Clitocybe squamulosa*

Color is the visual indicator used when judging the quality change of a foodstuff in the cooking process (35). It is the most intuitive indicator for evaluating the quality change in *C. squamulosa*. As shown in Table 3, the different heating principles, heating temperatures, and times of different cooking methods led to varying degrees of changes in the color characteristics of *C. squamulosa*. Specifically, boiled samples showed the highest brightness (L* value), and fried samples showed the lowest L* value, which may be attributed to the strong Maillard reaction that occurs during frying (36). The a* values of raw and cooked samples are all positive, indicating that mushrooms belong to the red variety. There is no significant difference between the boiled, steamed, and raw samples, and the microwave and fried samples were significantly lower (in terms of a* value) than the raw samples. The b* value observed in boiled and steamed samples is higher, and the b* value observed in microwave samples is closest to that of raw samples. The hue angle (*H*) is used to describe the changes in mushroom color, and values between 0 and 90 indicate that the color of *C. squamulosa* is between yellow, and the larger the value is, the more yellow the color (37). During the different cooking methods, the difference between the hue angle of the microwave and raw samples is minimized. Chroma (C*) is an index that describes the color intensity and visual characteristics related to color (22). The higher the saturation value, the brighter the color intensity. In contrast, boiling and steaming cooked samples have the highest C* values. The larger the a/b values (*h*) are, the darker the color is. Among them, the *h* values of fried samples are the highest, and there is no significant difference between steamed cooked and raw samples. The total color (Δ*E*) difference of the microwaved samples is the smallest.

**TABLE 3:** Effects of different cooking methods on color in *C. squamulosa.*

Cooking methods	L*	a*	b*	C*	*H*	*h*	△E*
CS	28.6 ± 0.76^d^	9.74 ± 1.19^a^	11.54 ± 1.53^b^	15.13 ± 1.69^b^	49.79 ± 3.54^d^	0.85 ± 0.10^a^	
BCS	36.86 ± 1.20^a^	9.17 ± 0.54^ab^	16.32 ± 1.65^a^	18.73 ± 1.51^a^	60.57 ± 2.65^a^	0.57 ± 0.06^d^	9.74 ± 1.76^a^
SCS	33.64 ± 1.02^b^	9.96 ± 1.06^a^	15.31 ± 1.17^a^	18.28 ± 1.41^a^	57.01 ± 2.20^b^	0.65 ± 0.06^c^	6.74 ± 1.48^b^
MFS	29.47 ± 0.60^c^	8.68 ± 0.99^b^	11.08 ± 1.44^b^	14.08 ± 1.68^c^	51.9 ± 1.89^c^	0.79 ± 0.05^b^	3.19 ± 1.42^c^
FCS	27.43 ± 1.09^e^	6.53 ± 0.73^c^	6.88 ± 0.76^c^	9.50 ± 0.95^d^	46.5 ± 2.64^e^	0.95 ± 0.09^a^	6.03 ± 2.35^b^

CS, *C. squamulosa*; BCS, boiling-cooked *C. squamulosa*; SCS, steaming-cooked *C. squamulosa*; MCS, microwaving-cooked *C. squamulosa*; FCS, frying-cooked *C. squamulosa*. Values represent the mean ± standard deviation, and different superscript lowercase letters denote significance (*p* < 0.05) in each column.

To summarize, the raw samples are yellowish-brown. The color of steamed and fried samples is darker, and the color of boiled samples is shown to be brighter. Microwave cooking can better maintain the primary color of *C. squamulosa*.

### Effects of cooking methods on conventional nutrients of *Clitocybe squamulosa*

Table 4 lists the effects of conventional nutrients of raw samples and cooking treatment on their nutritional value. The conventional nutrient contents of *C. squamulosa* are different after use of different cooking methods. The carbohydrate contents of the boiled and microwaved samples were higher than that of the steamed, microwave, and raw samples. Moreover, the loss of protein, ash, and moisture content of microwaved samples are the lowest, followed by the steamed samples. This may be because the steaming temperature is relatively low, and the microwave cooking is faster, so various chemical reaction rates and reaction times are reduced (38). The steamed and microwaved samples are not in direct contact with water or oil, which also reduces the loss of protein, ash, and water. Boiled and fried samples have suffered significant loss of nutrients, which may be related to water or oil absorption during boiling or frying, which has a dilutive effect on other nutrients (39). It is worth noting that the temperature can be as high as 180°C during frying, leading to severe oxidation of protein and fat and deterioration of mushroom quality (40). As the heating temperature of the water boiling, steaming, and microwave cooking methods is low, excessive oxidation can be avoided.

**TABLE 4:** Effects of different cooking methods on conventional nutrient components in *C. squamulosa.*

Nutrient component	CS	BCS	SCS	MCS	FCS
Energy (kJ/100 g)	1536.28 ± 3.82^c^	1.467.26 ± 4.99^e^	1518.65 ± 1.25^d^	1553.34 ± 4.55^b^	1890.03 ± 4.82^a^
Protein (g/100 g)	39.56 ± 0.16^a^	34.68 ± 0.44^c^	37.1 ± 0.16^b^	37.69 ± 0.75^b^	30.35 ± 0.99^d^
Fat (g/100 g)	9.70 ± 0.24^b^	7.74 ± 0.35^d^	8.89 ± 0.07^c^	9.08 ± 0.09^c^	22.93 ± 0.32^a^
Carbohydrate (g/100 g)	29.34 ± 0.45^d^	34.78 ± 0.12^a^	32.87 ± 0.17^b^	33.92 ± 0.98^ab^	30.63 ± 0.92^c^
Ash (g/100 g)	10.69 ± 0.20^a^	9.09 ± 0.12^b^	10.44 ± 0.13^a^	9.37 ± 0.19^b^	6.97 ± 0.25^c^
Moisture (g/100 g)	10.40 ± 0.27^b^	13.81 ± 0.29^a^	10.67 ± 0.06^b^	9.90 ± 0.27^c^	9.06 ± 0.25^d^

CS, *C. squamulosa*; BCS, boiling-cooked *C. squamulosa*; SCS, steaming-cooked *C. squamulosa*; MCS, microwaving-cooked *C. squamulosa*; FCS, frying-cooked *C. squamulosa*. Values represent the mean ± standard deviation, and different superscript lowercase letters denote significance (*p* < 0.05) in each row.

### Effects of cooking methods on the hydrolytic amino acids in *Clitocybe squamulosa*

The nutritional value and flavor of edible fungi are closely related to the type and content of amino acids, especially essential amino acids and flavoring amino acids (41). The differences in hydrolytic amino acid content in *C. squamulosa* with different cooking methods are listed in Table 5; in raw samples and cooked samples, the Arg content was significantly higher, followed by Glu and Asp, which together represent 32.64, 33.37, 32.42, 31.80, and 32.58% of the total amino acids in raw samples, boiled, steamed, microwaved, and fried samples. Among them, Arg is a functional essential amino acid that plays a vital role in hormone release, neurotransmission and maintenance of blood pressure, cell division and wound healing (42). In addition, Glu, Asp, Phe, Ala, Gly, and Tyr are all aromatic amino acids, which together represent 36.26, 37.40, 36.48, 36.02, and 37.11% of the total amino acids in raw samples, boiling, steaming, microwaved and frying cooked samples. These amino acids affect the flavor of *C. squamulosa*.

**TABLE 5:** Effects of different cooking methods on the hydrolytic amino acids in *C. squamulosa.*

Amino acid (g/100 g)	CS	BCS	SCS	MCS	FCS
Thr	1.69 ± 0.08^a^	1.51 ± 0.00^b^	1.60 ± 0.05^ab^	1.36 ± 0.05^c^	1.30 ± 0.06^c^
Val	1.84 ± 0.19^a^	1.54 ± 0.03^cd^	1.78 ± 0.13^ab^	1.59 ± 0.14^bc^	1.34 ± 0.02^d^
Met	0.67 ± 0.01^a^	0.58 ± 0.01^c^	0.62 ± 0.02^b^	0.57 ± 0.03^c^	0.53 ± 0.01^d^
Ile	1.58 ± 0.17^a^	1.39 ± 0.06^ab^	1.48 ± 0.12^a^	1.35 ± 0.16^ab^	1.17 ± 0.04^b^
Leu	2.44 ± 0.24^a^	2.09 ± 0.06^ab^	2.29 ± 0.20^ab^	2.02 ± 0.24^b^	1.56 ± 0.21^c^
Phe	1.22 ± 0.04^a^	1.25 ± 0.04^a^	1.25 ± 0.09^a^	1.10 ± 0.09^b^	1.13 ± 0.01^ab^
Lys	2.02 ± 0.08^a^	1.99 ± 0.02^a^	1.99 ± 0.09^a^	1.81 ± 0.26^ab^	1.69 ± 0.06^b^
Asp	2.85 ± 0.06^a^	2.61 ± 0.02^c^	2.74 ± 0.03^b^	2.27 ± 0.05^d^	2.17 ± 0.04^f^
Ser	1.55 ± 0.05^a^	1.36 ± 0.02^b^	1.47 ± 0.05^a^	1.30 ± 0.02^bc^	1.23 ± 0.07^c^
Glu	2.99 ± 0.02^a^	3.05 ± 0.09^a^	2.91 ± 0.09^a^	2.64 ± 0.30^b^	2.43 ± 0.07^b^
Gly	1.93 ± 0.11^a^	1.73 ± 0.04^b^	1.87 ± 0.02^a^	1.62 ± 0.01^c^	1.46 ± 0.04^d^
Ala	1.72 ± 0.05^a^	1.58 ± 0.02^c^	1.65 ± 0.02^ab^	1.46 ± 0.06^d^	1.33 ± 0.07^e^
Cys	0.88 ± 0.05^a^	0.86 ± 0.00^ab^	0.89 ± 0.02^a^	0.87 ± 0.01^ab^	0.83 ± 0.00^b^
Tyr	1.21 ± 0.05^a^	1.00 ± 0.01^bc^	1.17 ± 0.06^a^	1.07 ± 0.07^b^	0.96 ± 0.01^c^
His	1.13 ± 0.04^a^	1.04 ± 0.02^b^	1.10 ± 0.01^a^	0.99 ± 0.02^c^	0.90 ± 0.01^d^
Arg	4.89 ± 0.20^a^	4.23 ± 0.10^b^	4.65 ± 0.02^a^	4.06 ± 0.04^b^	3.73 ± 0.21^c^
Pro	2.27 ± 0.01^a^	2.18 ± 0.03^b^	2.30 ± 0.03^a^	2.11 ± 0.03^c^	1.79 ± 0.02^d^
Essential amino acids (EAA)	11.45	10.34	11.02	9.81	8.72
Non-essential amino acids (NEAA)	21.41	19.63	20.76	18.40	16.84
Umami amino acids (UAA)	11.92	11.21	11.59	10.16	9.49
Total amino acids (TAA)	32.87	29.97	31.77	28.21	25.57

CS, *C. squamulosa*; BCS, boiling-cooked *C. squamulosa*; SCS, steaming-cooked *C. squamulosa*; MCS, microwaving-cooked *C. squamulosa*; FCS, frying-cooked *C. squamulosa*. Values represent the mean ± standard deviation, and different superscript lowercase letters denote significance (*p* < 0.05) in each row.

Cooking temperature and time affect the rate of protein degradation, resulting in changes in the amino acid content (43). Compared with the raw samples, all cooking treatments resulted from decreased amino acid contents, and the degree of loss caused by different cooking methods was different. In terms of the total amount of amino acids, the order of their content is: raw > steamed > boiled > microwave > fried. Fried samples lost the most, which was possibly due to increasing degree of oxidation caused by the high temperature and oil, followed by microwaved and boiled samples. Microwave cooking is prone to thermal degradation, deformation, polymerization, and other reactions, leading to the loss of amino acids from the food (44). Boiled cooking readily causes a loss of amino acids in the soup. Thus, steamed cooking preserves the content of amino acid better than the other three cooking methods.

### Effects of cooking methods on bioactive compounds of *Clitocybe squamulosa*

The effects of cooking methods on the bioactive compounds of *C. squamulosa* are listed in Table 6. The polysaccharide content is shown to be higher, and the total phenol and flavonoid contents are lower. However, the four cooking treatments all decrease the amounts of bioactive compounds therein, but the extent of this loss is different. The difference in cooking conditions of all samples is undoubtedly the main reason for the difference in bioactive compound content. Among the four cooking methods, fried cooking lost the most, followed by boiled cooking. Steam cooking demonstrated a minimal loss of polysaccharides and microwave cooking showed minimal losses of total phenolics and flavonoids. The reason may be that different degrees of damage to the cell wall are caused by different heat-transfer media and cooking temperatures (45). For example, during the boiling process, the water content increases, the tissue was softened to make it easy for water-soluble compounds to dissolve in the soup (46). During the frying process, the oil covers the cell surface, hindering the extraction of bioactive compounds (46).

**TABLE 6:** Effects of different cooking methods on bioactive compounds contents in *C. squamulosa.*

Bioactive compounds	CS	BCS	SCS	MCS	FCS
Polysaccharide (mg/g)	129.76 ± 0.67^a^	91.46 ± 0.88^d^	106.78 ± 0.88^b^	101.45 ± 0.67^c^	62.93 ± 0.58^e^
Total phenolic (mg/g)	4.29 ± 0.28^a^	3.56 ± 0.01^d^	4.11 ± 0.01^c^	4.17 ± 0.02^b^	3.47 ± 0.03^e^
Flavonoids (mg/g)	15.00 ± 0.28^a^	13.78 ± 0.01^b^	14.55 ± 0.01^b^	14.94 ± 0.02^b^	12.69 ± 0.03^c^

CS, *C. squamulosa*; BCS, boiling-cooked *C. squamulosa*; SCS, steaming-cooked *C. squamulosa*; MCS, microwaving-cooked *C. squamulosa*; FCS, frying-cooked *C. squamulosa*. Values represent the mean ± standard deviation, and different superscript lowercase letters denote significance (*p* < 0.05) in each row.

### Effects of cooking methods on the antioxidant activity in *Clitocybe squamulosa*

**FIGURE 2:**
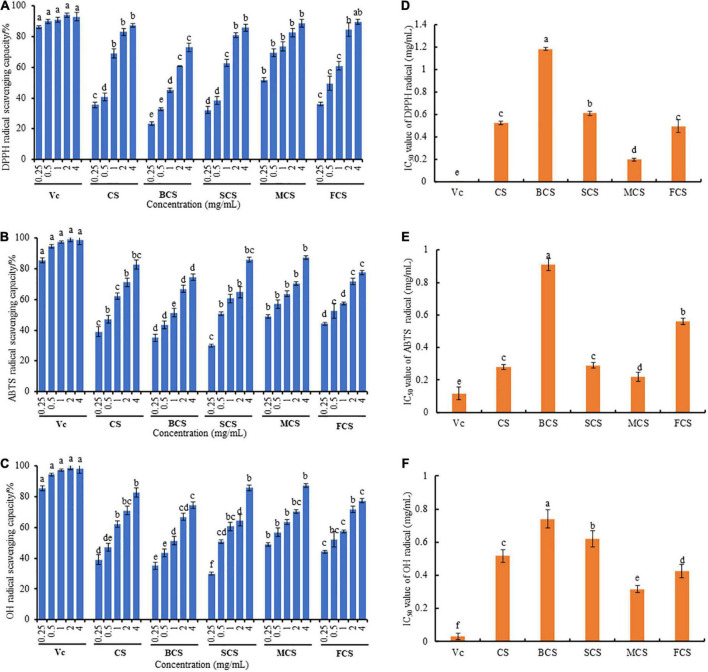
Effects of cooking methods on antioxidant activity of extracts from *C. squamulosa*. **(A)** DPPH radical scavenging activity. **(B)** ABTS radical scavenging activity. **(C)** OH radical scavenging activity. **(D)** IC_50_ value of DPPH radical. **(E)** IC_50_ value of ABTS radical. **(F)** IC_50_ value of OH radical. CS, *C. squamulosa*; BCS, boiling-cooked *C. squamulosa*; SCS, steaming-cooked *C. squamulosa*; MCS, microwaving-cooked *C. squamulosa*; FCS, frying-cooked *C. squamulosa*. Each value represents the mean ± SD (*n* = 3) and different lowercase letters denote statistical significance (*p* < 0.05).

The effects of cooking methods on the antioxidant activity of *C. squamulosa* were evaluated by DPPH, ABTS, and OH radical scavenging capacity (Figures 2A–F). The antioxidant activities for all measured concentrations of *C. squamulosa* extracts were dose-dependent, however, with a different extent. The DPPH, ABTS, and OH radical scavenging activities of the microwaved sample extract were greater than those of the raw sample and the other three cooked samples, but lower than that of the positive control (Vc). The IC_50_ values of the DPPH, ABTS, and OH radical scavenging activities of the microwaved sample were 0.20, 0.22, and 0.32 mg/mL, respectively. Compared with other cooking methods, microwave-cooked samples exhibited superior antioxidant activity, which may be attributed to the high contents of total phenols, polysaccharides, and flavonoids in microwaved samples. Of course, these were related not only to the content of bioactive components in mushrooms, but also to other components related to antioxidant activity therein (47). The IC_50_ values of ABTS radical scavenging activities between the steamed samples and raw samples were not statistically significant (*p* < 0.05). The IC_50_ values of the OH radical scavenging activities of the fried samples were significantly lower than that of the raw samples. Previous studies have found a positive correlation between bioactive compounds and antioxidant activity (48), therefore, after cooking, the enhanced antioxidant activity of *C. squamulosa* may be ascribed to the destruction of covalent bonds during heating, allowing cells to release more active ingredients (49).

## Conclusion

The effects of different cooking methods on the volatile flavor compounds and nutritional constituents, and antioxidant activities of *C. squamulosa* were investigated by boiling, steaming, microwaving, and frying. From the perspective of volatile compounds, after cooking treatment, the lipids of *C. squamulosa* were lost to a significant extent, but new alcohol and acid compounds were produced, and the quantity and content of alcohols, aldehydes and ketones increased. Based on ROAV values and PCA, steaming and microwave cooking can improve the flavor of *C. squamulosa*. From a color difference and nutritional content perspective, each cooking treatment resulted in positive color changes, with microwave cooking closest to the color of raw samples. Among all samples, steamed and microwaved *C. squamulosa* had a higher nutrient content. An antioxidant capacity analysis found that microwave-cooked samples showed the highest antioxidant activity. Comprehensive analysis found that steaming and microwave cooking were more suitable for cooking *C. squamulosa*.

In the diet, the nutritional value of food is not only related to how it is cooked, but also related to the digestion and absorption of nutrients by the body. During digestion, the nutritional value contained in the food is transmitted to the parts needed by the body, providing energy for the human body and maintaining health. Exploring the effects of different cooking methods on the nutritional value and volatile flavor of food is crucial to basic research in the early stage. In the later stage, attention should also be paid to the degree of digestion and absorption of food nutrients in the human body after use of different cooking methods. Therefore, the further to explore the digestion and absorption of *C. squamulosa* by the human body during the digestion after use of different cooking methods, the samples should also be subjected to *in vitro* simulated digestion tests. Then, the effects of different cooking methods on the quality of *C. squamulosa* were comprehensively evaluated. This provides a theoretical basis for people to choose appropriate cooking methods and provides a reference for the cooking and processing of other edible fungi.

## Data availability statement

The original contributions presented in this study are included in the article/supplementary material, further inquiries can be directed to the corresponding authors.

## Author contributions

HY: conceptualization, methodology, formal analysis, data curation, and writing-original draft. ZL: data curation and software. LX: resources, methodology, investigation, and writing-review. MC: resources and funding acquisition. JM, CF, XG, and YC: resources, conceptualization, formal analysis, and supervision. All authors contributed to the article and approved the submitted version.
